# Age‐related impairment of declarative memory: linking memorization of temporal associations to GluN2B redistribution in dorsal CA1

**DOI:** 10.1111/acel.13243

**Published:** 2020-10-03

**Authors:** Alice Shaam Al Abed, Azza Sellami, Mylene Potier, Eva‐Gunnel Ducourneau, Pauline Gerbeaud‐Lassau, Laurent Brayda‐Bruno, Valerie Lamothe, Nathalie Sans, Aline Desmedt, Peter Vanhoutte, Catherine Bennetau‐Pelissero, Pierre Trifilieff, Aline Marighetto

**Affiliations:** ^1^ INSERM Neurocentre Magendie Bordeaux France; ^2^ Neurocentre Magendie Bordeaux University Bordeaux France; ^3^ Bordeaux Sciences Agro Bordeaux France; ^4^ Institute of Biology Paris Seine INSERM UMR‐S1130 Neuroscience Paris Seine Paris France; ^5^ CNRS UMR 8246 Neuroscience Paris Seine Paris France; ^6^ UPMC Université Paris 06 UM CR18 Neuroscience Paris Seine Sorbonne Université Paris France; ^7^ INRAE Bordeaux INP NutriNeuro Bordeaux University Bordeaux France; ^8^ Present address: Eccles Institute of Neuroscience John Curtin School of Medical Research The Australian National University Canberra ACT Australia

## Abstract

GluN2B subunits of NMDA receptors have been proposed as a target for treating age‐related memory decline. They are indeed considered as crucial for hippocampal synaptic plasticity and hippocampus‐dependent memory formation, which are both altered in aging. Because a synaptic enrichment in GluN2B is associated with hippocampal LTP in vitro, a similar mechanism is expected to occur during memory formation. We show instead that a reduction of GluN2B synaptic localization induced by a single‐session learning in dorsal CA1 apical dendrites is predictive of efficient memorization of a temporal association. Furthermore, synaptic accumulation of GluN2B, rather than insufficient synaptic localization of these subunits, is causally involved in the age‐related impairment of memory. These challenging data identify extra‐synaptic redistribution of GluN2B‐containing NMDAR induced by learning as a molecular signature of memory formation and indicate that modulating GluN2B synaptic localization might represent a useful therapeutic strategy in cognitive aging.

## Graphical abstract

Memorizing temporal association is associated with an extra‐synaptic redistribution of GluN2b‐containing NMDA receptors in young adult mice. A loss of such trafficking, leading to GluN2b synaptic accumulation, causes the age‐related memory deficit. Indeed, reducing GluN2b synaptic contents by inducing extra‐synaptic redistribution of these subunits with a peptide interfering with their anchorage at the PSD (TAT‐GluN2b‐9c) or with an estradiol (E2)‐treatment, rescues temporal memory in aged mice.

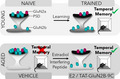

## 1 INTRODUCTION

The episodic component of declarative memory (DM), that is, our capability to remember conjunctions of *what happened where and when*, undergoes preferential degradation during aging. Memorizing temporal and spatial/contextual associations that underlie DM is known to critically rely on the hippocampus (Eichenbaum & Cohen, 2014), but mechanisms at the cellular/molecular level remain largely hypothetical. A strong candidate mechanism is glutamate *N*‐methyl‐d‐aspartate receptors (NMDAR)‐dependent synaptic plasticity (Baudry et al., 2015; Nicoll, 2017) under the form of long‐term potentiation (LTP) at hippocampal CA1 synapses (Huerta et al., 2000; Tsien et al., 1996; but see (Taylor et al., 2014) for a conflicting view).

Even though LTP has been extensively studied (Paoletti et al., 2013; Shipton & Paulsen, 2014; Yashiro & Philpot, 2008), it is only recently that NMDAR lateral membrane trafficking between synaptic and extra‐synaptic sites has been discovered as a main mechanism for synaptic plasticity (Bellone & Nicoll, 2007; Dupuis et al., 2014; Kellermayer et al., 2018; Potier et al., 2016). In particular, a rapid rearrangement of GluN2A and GluN2B subunits‐containing NMDAR, leading to a synaptic enrichment in GluN2B, is associated with LTP in cultured hippocampal neurons (Bellone & Nicoll, 2007; Dupuis et al., 2014; Kellermayer et al., 2018). However, whether a redistribution of GluN2B‐containing NMDAR from extra‐synaptic to synaptic sites is effectively induced by learning in the hippocampus and causally contributes to DM formation remains to be established.

Here we directly tackled this question in mice by focusing on a crucial component of declarative memory formation: the dorsal (d)CA1‐dependent memorization of temporal associations, which is degraded in aging (Sellami et al., 2017). We first measured synaptic localization of GluN2 subunits by visualizing their proximity to the post‐synaptic density (PSD) protein, PSD‐95 using a proximity ligation assay (PLA; Alsemarz et al., 2018; Trifilieff et al., 2011). We focused on the apical dendrites of dCA1 since we had previously identified a NMDAR surface trafficking‐dependent LTP in this area (Potier et al., 2016). We then manipulated synaptic localization of GluN2 subunits by modulating their interaction with PSD‐95 (Niethammer et al., 1996) using a TAT interfering peptide (Aarts et al., 2002). These correlative and interventional molecular approaches were combined to behavioral assessments of memory for temporal associations in young and aged mice.

## 2 RESULTS

### 2.1 Learning‐induced reduction of GluN2B synaptic localization correlates with the memorization of a temporal association

**FIGURE 1:**
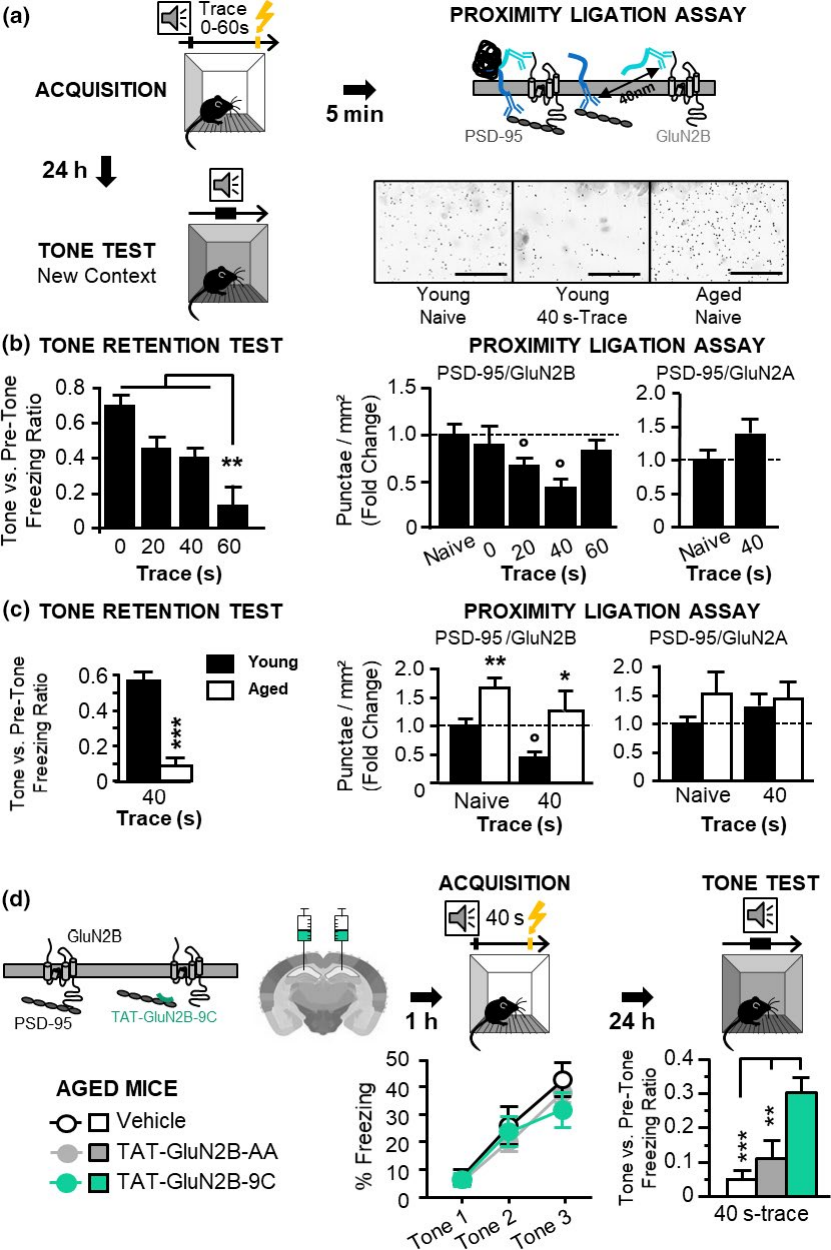
Learning‐induced reduction of synaptic GluN2B in dorsal CA1 apical dendrites correlates with efficient temporal binding (TB)‐dependent memorization and synaptic accumulation of GluN2B‐containing NMDAR contributes to the age‐related impairment of TB‐dependent memory. (a) correlative approach protocol: young and aged mice were submitted to the acquisition of (0‐60 s‐) TFC. These trained mice were either killed 5 min after TFC for measuring GluN2B/A synaptic densities (in comparison with naive controls) by proximity ligation assay, PLA in dorsal CA1 apical dendrites, or submitted to a Tone retention test 24 h later, to assess memory of the trace tone‐shock association, which requires TB, the longer the trace, the more the TB demand. Bottom right: Brightfield staining of the PLA in the apical dendrites of dCA1, representative of the level of GluN2B‐PSD95 signal in the young naive, young 40 s trace and aged naive conditions. Scale bar: 25 µm. (b) Behavioral (left) and PLA (right) results in young mice. Compared to naïve mice, TFC induces a diminution of synaptic GluN2B (but not GluN2A)‐NMDAR only when TB is required and efficient, that is, with 20 s‐ or 40 s‐trace, but not with 0 s = no‐trace or 60 s‐trace (beyond TB capability) [(Behavior: *Trace effect* on freezing ratio *F*_3,51_ = 9,39; *p* < 0.0001; post hoc PLSD: *p* < 0.0001, *p* = 0.003, *p* = 0.019 for 60 s‐trace vs 0 s‐, 20 s‐ and 40 s‐trace respectively, *N* = 13‐14 by group); (PLA of PSD95/GluN2B, *t* tests: *p* = 0.047 and *p* = 0.027 for naive vs 20 s‐trace and 40 s‐trace; Naive *N* = 16, Trained *N* = 6–10 by trace‐group)]. (c) Behavioral (left) and PLA (right) results in young and aged mice trained with a 40 s‐trace. In aged mice, the absence of memory for the 40 s‐trace association (despite normal acquisition of TFC, *cf* Figure S1b) attesting of impaired TB‐dependent memory is associated with increased contents of synaptic GluN2B (but not GluN2A)‐NMDAR and a lack of TFC‐induced reduction of these contents [(Behavior, *Age effect* on freezing ratio: *F*_1,21_ = 78.87, *p* < 0.001; *N* = 9/13 for young/aged); (PLA of PSD95/GluN2B, *t* tests: *p* = 0.0063 and *p* = 0.01 for young vs aged in naive and trained condition, respectively, and *p* = 0.016 for 40 s‐trace vs naive in young groups; Naive young/aged *n* = 11/9, Trained young/old *n* = 6/5)]. (d) interventional approach by intraCA1 infusion of TAT‐peptides before 40 s‐TFC in aged mice: only TAT‐GLUN2B‐9C, which leads to GluNR2B extra‐synaptic redistribution by interfering with PSD95/GluN2B (Aarts et al. 2002), improved TB‐dependent memory in aged mice (*Treatment effect* on freezing ratio *F*_2,27_ = 9.375; *p* < 0.0008; post hoc PLSD: *p* = 0.004 and *p* = 0.0005 for TAT‐9C vs TAT‐AA and vehicle, respectively; *N* = 10 by group). *p < 0.05, **p ≤ 0.01; ***p ≤ 0.001; open circles for PLA comparison with the naive condition. Data presented as mean ± *SEM*

A first necessary step was to demonstrate that learning induces changes in GluN2A/B synaptic contents in dCA1 apical dendrites. GluN2A/B synaptic localization, revealed through its proximity with the synaptic scaffolding protein PSD‐95, was measured by PLA (Figure 1a) immediately after the acquisition of *auditory trace fear conditioning* in young adult mice. This one‐trial learning task requires memorizing an association between 2 temporally discontiguous stimuli, a process called temporal binding (TB), which is crucial for DM formation and depends on dCA1 activity (Sellami et al., 2017). During acquisition, a tone (conditioned stimulus, CS) is paired with a mild electric foot‐shock (unconditioned stimulus, US), separated by a brief time interval: *the trace*, thus making the CS and US discontiguous. Mnemonic retention of the tone‐shock association hence becomes dependent of TB: the longer the trace, the more the TB demand. In order to modulate this TB demand, we trained 4 groups of young adult mice using different trace intervals (0 s = no‐trace, 20 s, 40 s, or 60 s‐trace). One part of each trained group was killed immediately after learning for PLA analysis, while the other part performed a retention test 24 h later in order to assess TB‐dependent memory. Retention of the tone‐shock association was measured by the fear response (freezing) elicited by re‐exposure to the tone alone in a neutral context (i.e., *conditioned fear response*). Behavioral results confirmed our previous findings (Sellami et al., 2017) that TB efficiency declines with increased trace interval and is limited to <60 s intervals in young mice (Figure 1b‐left, Figure S1a). PLA results revealed that the acquisition of trace fear conditioning induces a reduction of synaptic GluN2B‐containing NMDAR correlating with TB function. Indeed, this decrease of synaptic content occurred as long as TB was required and efficient, *that is*, for the 20 s‐ and 40 s‐trace conditions, but not in the group trained with a 0 s‐trace (no TB demand), nor in the group trained with a 60 s‐trace (above TB capability; Figure 1b‐middle, see Figure S1b–f for control experiments of signal specificity). The diminution of synaptic expression was specific of GluN2B subunits since synaptic GluN2A levels remained unchanged (Figure 1b‐right). Thus, a redistribution of GluN2B‐containing NMDAR to extra‐synaptic sites is associated with efficient storage of temporal association. Such learning‐induced rearrangement of NMDAR differs from the one associated with LTP in vitro (Bellone & Nicoll, 2007; Dupuis et al., 2014; Kellermayer et al., 2018).

### 2.2 Synaptic accumulation of GluN2B‐containing NMDAR as a molecular signature of age‐related impairment of temporal binding (TB)‐dependent memory

We then examined whether an alteration of the learning‐induced redistribution of GluN2B was involved in the age‐related degradation of TB‐dependent memory, using 40 s‐trace tone fear conditioning (Sellami et al., 2017). Similarly to what we previously found (Sellami et al., 2017), aged mice showed normal acquisition of the task (Figure S1g left panel) but no retention of the trace association 24 hours later (Figure 1c‐left; Figure S1 g right panel), a profile attesting of their TB‐dependent memory impairment. PLA analyses revealed that the synaptic content of GluN2B‐, but not GluN2A‐containing NMDAR, was increased in aged mice relative to young ones, in both naive and trained conditions (Figure 1c‐middle). Moreover, acquisition of 40 s‐trace conditioning (Figure 1c middle, and of 20 s‐trace conditioning, Figure S1h) failed to decrease GluN2B/PSD95 complexes in aged animals. Since the expression of GluN2A/B or PSD‐95 remained unchanged with aging (Figure S1i), these findings are indicative of a specific alteration of the synaptic trafficking of GluN2B‐containing NMDAR.

### 2.3 Interfering peptide reducing GluN2B synaptic levels alleviates the aging‐related memory impairment

To assess whether the alteration of GluN2B‐containing NMDAR trafficking was causally linked to the age‐related memory impairment, we used the cell‐permeable TAT‐GluN2B‐9c peptide, which decreases GluN2B‐containing NMDAR synaptic content by disrupting GluN2B/PSD95 interaction (Aarts et al., 2002; Gardoni et al., 2009), as confirmed here by PLA (Figure S2). Bilateral intra‐dCA1 infusions of the TAT‐GluN2B‐9C peptide, or the TAT‐GluN2B‐AA control peptide that cannot interfere with PSD95/GluN2B (Aarts et al., 2002), were performed 1 h before PLA or the acquisition of 40 s‐trace fear conditioning in aged mice. Molecular results confirmed a (≈90%) reduction of local GluN2B synaptic contents (with no change in GluN2A) after treatment with the TAT‐GluN2B‐9C peptide (Figure S2). Behaviorally, while all groups displayed similar acquisition of the trace conditioning, only the TAT‐GluN2B‐9C‐injected group displayed a significant retention of the tone‐shock association, indicative of efficient TB‐dependent memorization (Figure 1d). Thus, synaptic accumulation of GluN2B‐containing NMDAR is causally involved in the age‐related memory impairment.

### 2.4 Concomitant reduction of GluN2B synaptic levels and improvements of CA1 activity‐dependent temporal binding by 17β‐estradiol (E2) supplementation in aged mice

To further assess whether reducing GluN2B synaptic accumulation could represent a valuable strategy against age‐related cognitive degradation, we studied the effects of E2 for two main reasons. First, in cultured hippocampal neurons, E2 alters surface trafficking of GluN2B‐containing NMDAR resulting in significant reduction of their synaptic location after long‐term (24 h) exposure (Potier et al., 2016). Second, in aged (male) mice, E2 improves the retention of hippocampus‐dependent memory when chronically administered through drinking water (1 µM; Al Abed et al., 2016). We examined whether such treatment could rescue TB‐dependent memory in aged mice through an impact on NMDAR synaptic distribution.

**FIGURE 2:**
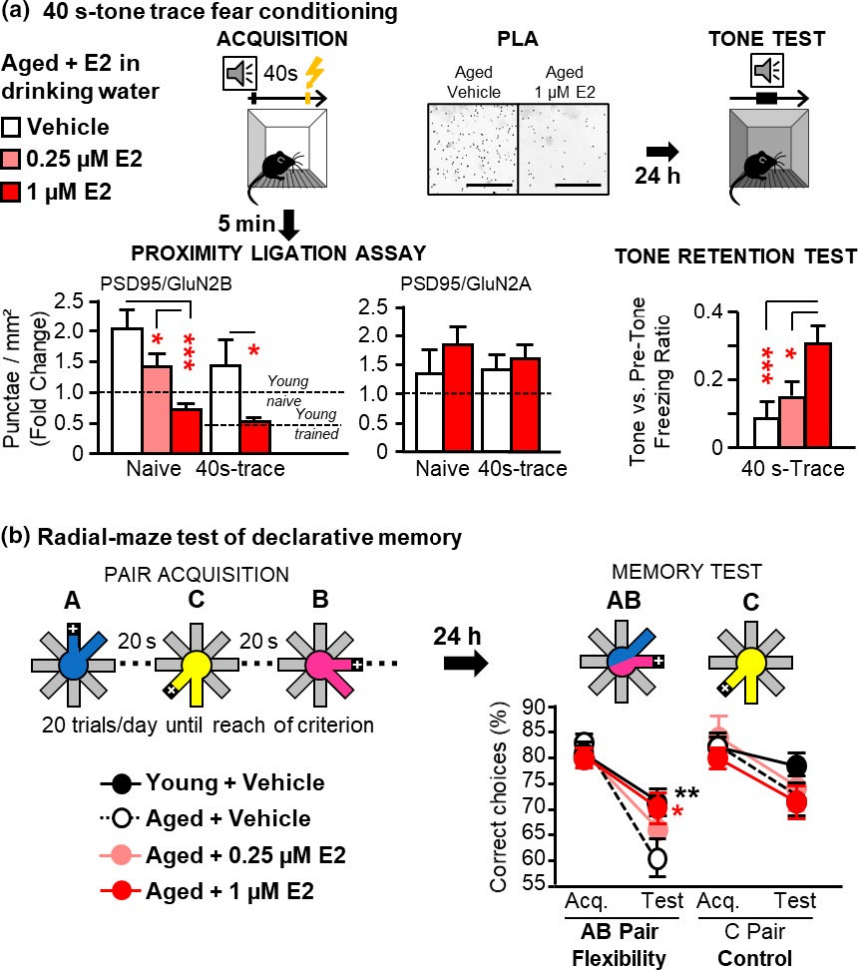
Concomitant dose‐dependent reduction of GluN2B synaptic levels and improvement of temporal binding (TB)‐dependent memory by drinking water 17β‐estradiol (E2) supplementation in aged mice (a) The same correlative approach as in Figure 1, that is Trace‐tone fear conditioning, TFC combined to PLA in dCA1 apical dendrites, showing that the dose which normalizes GluN2B synaptic levels to young (naive and trained) levels (1 µM E2), also enhances the 24 h‐retention of 40 s‐TFC attesting of the restoration of TB‐dependent memory in aged mice. *Left panel*:* Dose effect of E2 treatment on* PLA. E2 at the 0.25 µM dose only partially reduced the PLA signal in aged naive mice (0.25 µM vs. vehicle: ns), while the dose of 1 µM normalized PLA signal to young levels in both naive and trained conditions (1 µM vs vehicle: *p* = 0.0003 and *p* = 0.0103, respectively; *n* = 6‐10 per group). *Right panel*: Dose effect of E2 treatment on the 24 h‐retention of TFC under a 40 s‐trace. The E2 treatment alleviated the age‐related deficit of retention of trace conditioning at the 1 µM dose only (*E2 Effect in Aged mice*: *F*_2,28_ = 8.51, *p* = 0.0013; Veh. vs. E2 0.25 µM: ns; Veh. vs. E2 1 µM: *p* = 0.0003; n = 8‐12 per group). (b) Dose effect of E2 supplementation in the radial maze model of declarative memory degradation in aged mice confirming the cognitive benefit of 1 µM E2: *In the acquisition phase*, groups of young (*N* = 11), aged vehicle (*N* = 10) or (0.25/1 µM *N* = 6/8) E2‐treated mice were trained to choose the food‐rewarded (+) arm of each unvarying pair (A, B, C) and reached similar levels of accuracy (*see* also Figure S3c acquisition curves for the 3 groups). However, *in the test phase* when AB pairs were recombined to assess characteristic flexibility of declarative memory (Pair C was unchanged serving as control), untreated aged mice performed at about chance level, exhibiting poorer performance than young controls (For AB pair: *Evolution Acquisition vs test X Age in Vehicle*: *F*_1,19_ = 6.649; *p* = 0.0184; *Age effect for recombined AB pair*: *F*_1,19_ = 9.115; *p* = 0.0071), and this performance was restored by the 1 µM dose only *(Recombined pair AB*:* Vehicle* vs. 1 µM* E2 in Aged*: *p* = 0.0309; *Vehicle vs*.* 0*.*25 µM in Aged*: *p* = 0.08 ns). *p < 0.05; **p ≤ 0.01; ***p ≤ 0.001; red asterisk represent the E2 effect in aged mice. Data presented as mean ± *SEM*

First, we used the 40 s‐trace conditioning task in combination with PLA. Here again, aged mice acquired the conditioning task regardless of their treatment group (Vehicle, 0.25, or 1 µM of E2 in drinking water; Figure S3a). However, only those treated with the highest dose of E2 showed a 24 h‐retention of the temporal association (Figure 2a right panel). Such memory restoration was associated with a reduction of GluN2B synaptic content to levels comparable to those of young mice, without any change in GluN2A synaptic content (Figure 2a left panel), or GluN2A/B and PSD‐95 protein levels (Figure S3b).

**FIGURE 3:**
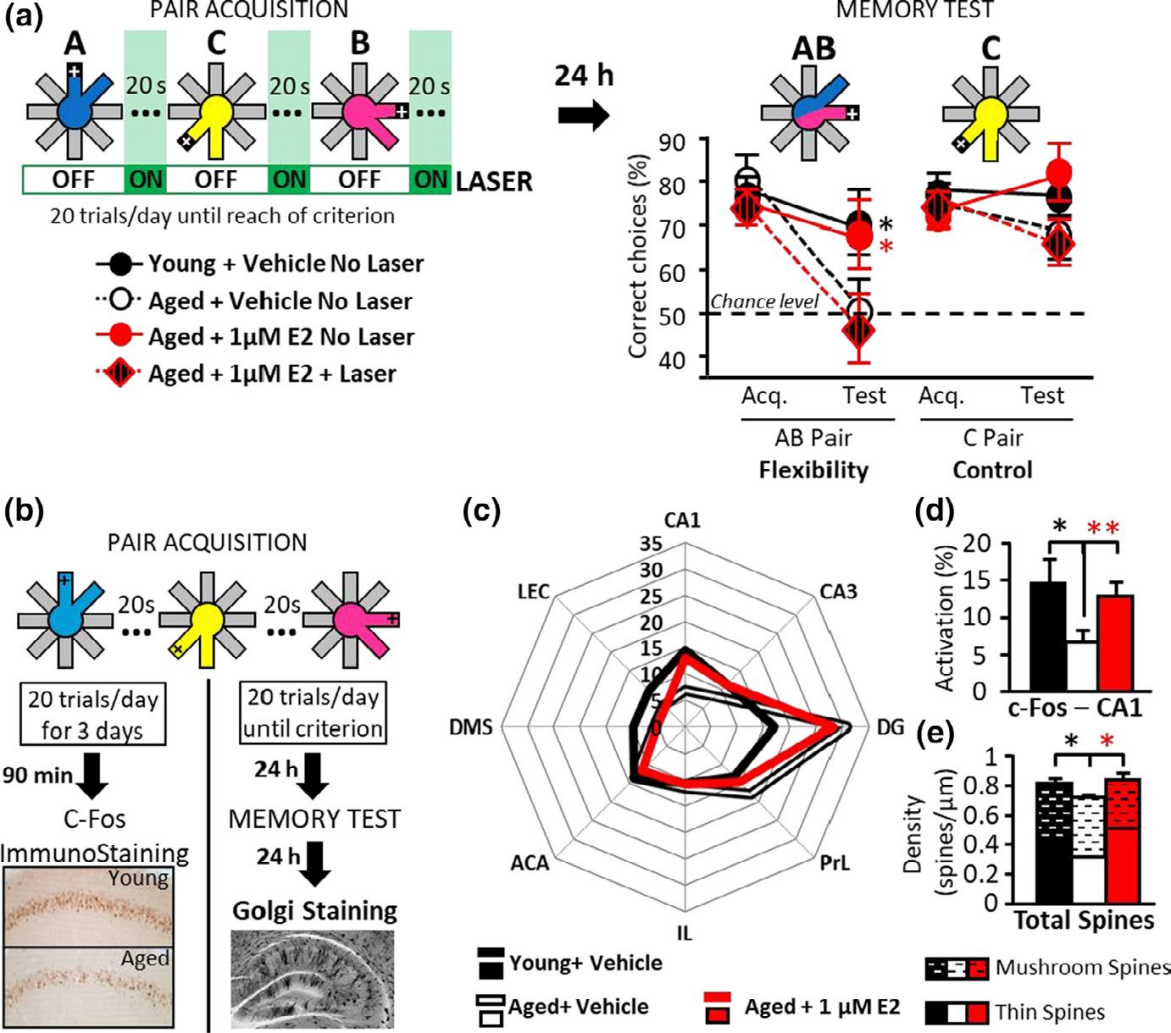
17β‐estradiol supplementation restores age‐related impairment of declarative memory by normalizing CA1 function in temporal binding (TB). (a) Optogenetic inhibition of CA1 during the inter‐pair intervals of the acquisition phase (only) prevents the memory benefit of (1 µM) E2. The same radial maze task was used as in Figure 2b, except that the CA1 of mice treated with (1 µM) E2 was optogenetically inhibited during the 20 s‐ inter‐pair intervals of the acquisition stage to study potential implication of CA1 cells’ activity across temporal intervals between events,^6^ which sustains temporal binding (TB). As in the previous experiment (Figure 2b), all groups performed equally well whichever the pair at the end of the acquisition stage, and E2 (1 µM) improved the performance of aged mice in the subsequent test of memory flexibility (for AB pair: *Evolution Acquisition vs Test X Age* in Vehicle *F*_1,14_ = 6.127; *p* = 0.0267; *Evolution Acquisition vs Test X Treatment in Aged No laser groups* *F*_1,13_ = 8.672; *p* = 0.0114; *E2 effect in aged No laser groups for AB pair*: *F*_1,13_ = 4.477; *p* = 0.0484). In contrast, E2‐treated mice that have acquired the initial 3 pairs under optogenetic inhibition of CA1 cells during the inter‐pair intervals performed as poorly as vehicle‐treated mice in the flexibility test (*Laser effect* in *E2*‐* treated aged mice*: *F*_1,10_ = 8.306; *p* = 0.0163). Thus, the rescue of declarative memory by E2 is through the improvement of CA1 function of TB. *N* = 6‐9/group. (b–d) 1 µM E2 normalizes CA1 Fos activation and spine morphology. (b) Protocol. For c‐Fos analyses, groups of young + vehicle, aged + vehicle and aged + 1 µM E2 mice (*n* = 6, 13, 10, respectively) were prepared for immunohistochemistry after the 3rd training session of the acquisition phase of the radial maze task. For Golgi analyses, all groups (*n* = 4 young + vehicle, *n* = 3 aged + vehicle, and *n* = 3 aged + 1 µM E2) underwent both stages of the radial maze task and were prepared 24 h later for Golgi staining. (c) Effects of age and treatment on the learning‐induced activation of an ensemble of memory‐related areas. c‐Fos positive cells were counted in the dorsal hippocampus (dCA1, dCA3, Dentate Gyrus, dDG), pre‐frontal cortex (Pre‐Limbic, PrL and Infra‐Limbic, IL), Anterior Cingulate Area (ACA); Dorso‐Medial Striatum (DMS) and Lateral Entorhinal Cortex (LEC). Here, c‐Fos activation was expressed as a percentage of activation for each area relative to the total activation (i.e., sum of studied areas). This relative level of activation was modified for certain structures only in the aged + vehicle group compared to the young one. Namely, there was less activation for CA1 (*t* test: *p* = 0.0188) and DMS (*p* = 0.0031), but more activation for DG (*p* = 0.0033). Remarkably, CA1 was the only structure of the network in which E2 supplementation had an effect (*t* test: *p* = 0.0065). (d) Effects of age and treatment on the total number of spines per µm of dendritic shaft and proportion of thin spines (plain histograms, bottom) and mushroom spines (dashed histograms, top) in dorsal CA1 apical dendrites. Compared to young mice, the aged + vehicle group displayed a significant reduction of spine density, which was normalized by 1 µM E2 treatment (*t* test: *Age effect in* vehicle groups: *p* = 0.0113; 1 µM E2 effect *in aged groups*: *p* = 0.048). Analyses of spine morphology showed that aging alters the spine profile. Indeed, aging had no effect on *mushrooms spine density* but significantly reduces *thin spine density* (*t* test: *Age*: *p* = 0.0185). 1 µM E2 restores a more youthful spine profile by significantly reducing mushrooms spines but increasing thin spines (*t* test: *Treatment*: *p* = 0.0382; *p* = 0.0177, respectively). *p < 0.05; **p ≤ 0.01; red asterisk represents the E2 effect in aged mice. Data presented as mean ± *SEM*

Second, we used our radial maze model of age‐related decline of relational/declarative memory (Etchamendy et al., 2012; Marighetto et al., 1999), which previously enabled us to demonstrate the critical implication of CA1‐dependent TB in DM formation (Sellami et al., 2017). In the acquisition phase of this task, aged mice were capable of learning to choose the rewarded side among each of three unvarying pairs of arms, repeatedly presented with a 20 s‐inter‐pair interval (Figure S3c). However, vehicle‐treated ones failed when the testing situation was modified by recombination of the pairs, which assesses mnemonic flexibility, a characteristic property of relational/declarative memory expression (flexibility test, Figure 2b). This flexibility deficit results from an incapability to associate the 20 s‐distant pairs of the acquisition phase into a relational mnemonic representation. In other words, the age‐related deficit originates from TB impairment, which is due to blunted dCA1 activity across the (20 s‐) inter‐pair intervals of the acquisition phase (Sellami et al., 2017). We here showed that mnemonic flexibility was improved by E2 at the sole dose capable of normalizing GluN2B synaptic contents in dCA1 (Figure 2a,b). We then demonstrated that this improvement was related to the restoration of dCA1 activity‐dependent TB function (Figure 3). Indeed, first, in aged mice expressing the inhibitory ArchaeRhodopsin (Sellami et al., 2018), we found that optogenetic silencing of CA1 cells during the 20 s‐inter‐pairs of the radial maze acquisition prevented the ability of E2 to alleviate the memory impairment (Figure 3a). Second, immunostaining of the cellular activity marker c‐Fos performed after acquisition of the radial maze task showed that E2 treatment increased c‐Fos expression only in dCA1, thus normalizing the relative weight of this structure in the network without affecting any other structure (Figure 3b–d). Third, E2 treatment also normalized thin and mushroom spines density in secondary apical dendrites of dCA1 pyramidal cells (Figure 3b‐right, Figure 3e).

In complement to above‐described findings with the TAT peptide, present effects of E2 at a dose that normalizes GluN2B synaptic levels in aged mice support the conclusion that synaptic accumulation of GluN2B contributes to the aging‐associated degradation of dCA1‐dependent TB leading to relational/declarative memory decline.

## 3 DISCUSSION

Our work unveils an unexpected trafficking of hippocampal NMDAR during memory formation by showing post‐learning rearrangements of GluN2A/B subunits that differ from the ones associated with LTP in vitro (Bellone & Nicoll, 2007; Dupuis et al., 2014; Kellermayer et al., 2018). Precisely, rather than a synaptic enrichment, an extra‐synaptic redistribution of GluN2B‐containing NMDAR is associated with successful memorization of temporal associations, that is, a crucial component of DM formation (Sellami et al., 2017). This finding challenges current ideas regarding the role of GluN2A/B subunit rearrangements, according to which a synaptic enrichment in GluN2B promotes new memory formation by facilitating synaptic LTP (Bellone & Nicoll, 2007; Dupuis et al., 2014; Kellermayer et al., 2018; Quinlan et al., 2004).

Regarding aging, the present study further reinforces our previous findings that impairment of dCA1‐dependent TB process is causally involved in the DM decline (Sellami et al., 2017). Herein, we unraveled a main mechanism involved in this decline, that is, an alteration of GluN2B‐containing NMDAR trafficking, which leads to synaptic accumulation of these receptors (see also Zamzow et al., 2013) in dCA1 apical dendrites of aged mice. We cannot exclude that part of the aging‐related increase of GluN2B/PSD95 PLA signal might be due to pre‐synaptically localized GluN2B‐containing NMDAR. Indeed, hippocampal pre‐synaptic contents of these receptors tend to increase with aging in ovariectomized rats (Waters et al., 2019). Another possible mechanism at play could be the altered functionality of GluN2B subunits, subsequent to an age‐related modification of oxidative stress metabolism (Kumar et al., 2019). Nevertheless, the memory enhancing effect of intra‐CA1 infusion of Tat‐NR2B‐9c peptide interfering with GluN2B‐PSD95 (Aarts et al., 2002; Gardoni et al., 2009), supports that the abnormal accumulation of GluN2B subunits at the PSD is causally involved in the age‐related cognitive impairment.

Our findings complement a previous study (Zamzow et al., 2013), showing that GluN2B association with membrane scaffolding proteins including PSD95 (but also extra‐synaptic GIPC) augments with aging in the hippocampus. Altogether, these findings are suggestive of an increase of GluN2B anchorage to membrane proteins that should impair GluN2B mobility. Similarly, pre‐frontal levels of phospho‐Tyr1472‐GluN2B (synaptic) measured in aged female monkeys were found to be inversely correlated with accuracy in a working memory task (Hara et al., 2018).

Causality presently established between synaptic accumulation of GluN2B and the degradation of hippocampal function in TB/DM, challenges the hypothesis that a reduction in GluN2B‐dependent transmission contributes to cognitive aging (e.g., Hara et al., 2018; Wang et al., 2014) and highlights the targeting of the receptor's mobility as a potential alternative therapeutic strategy. Herein we show that normalization of GluN2B subunits distribution in aged male mice by nutritional supplementation with E2 is associated with concomitant normalization of both spine morphology and TB‐related activity of dCA1, leading to TB and DM restoration. The mechanism by which E2 impacts synaptic dynamics of GluN2B‐NMDAR is unknown. Nevertheless, our results extend current literature on potential interest of estrogens for treating cognitive aging in both males and females (Bean et al., 2014; Frick, 2015; Lewis et al., 2008; Smith & McMahon, 2006; Vedder et al., 2013; Woolley & Mcewen, 1994).

Altogether, the present findings demonstrate that synaptic rearrangements of hippocampal GluN2B‐containing NMDAR occur during learning and sustain the formation of declarative memory, hence complementing our understanding of the physiological significance of NMDAR synaptic trafficking.

## 4 EXPERIMENTAL PROCEDURES

### 4.1 Animals

Studies were conducted using young (3–4 months) or aged (20–23 months) male C57/BL6j mice (Charles River). From their arrival, mice were collectively caged in animal standardized room conditions under a 12:12 light:dark cycle, and ad libitum food and water.

For estradiol treatment only, mice were caged in small groups (4‐7 mice per group) at the beginning of treatment and the food was replaced by a soybean‐less food (LASQCdiet^®^, Rod18, Rad).

For all experiments, 10–7 days before the beginning of the experiments, mice were caged separately.

Finally, for radial maze experiments only, mice were submitted to a partial food deprivation (85%–90% of their free feeding weight) throughout the duration of training.

All experiments were conducted in accordance with the European directive 2010‐63‐EU and with approval from the Bordeaux University Animal Care and Use Committee (N°5012035A‐N°1377). All efforts were made to minimize suffering and reduce the number of animals used.

### 4.2 Treatments with 17β‐Estradiol

Aged mice were supplemented with 17β‐Estradiol (E2; Sigma‐Aldrich E8875) in drinking water for two weeks prior to behavioral experiments. There were three treatment groups: Aged+Vehicle (water 2% Propanediol‐1,2 (Prolabo, PG), Aged+0.25 µM E2 (2% PG), and Aged+1 µM E2 (2% PG). Young mice received the Vehicle solution. From the beginning of the treatment, the food was replaced by a soybean‐less food (Harlan Teklad Global Diet; Harlan Laboratories Inc). Treatments lasted until sacrifice. We checked that all mice approximately drank the same amount of water (≈4 ml/day).

### 4.3 Trace fear conditioning task (TFC)

#### 4.3.1 Apparatus

Fear conditioning behavior was performed in a Plexiglas conditioning chamber (30 × 24 × 22 cm), in a brightness of 100 lux, given access to the different visual‐spatial cues in the experimental room. The floor of the chamber consisted of stainless‐steel rods connected to a shock generator (Imetronic).

#### 4.3.2 Trace fear conditioning procedure

The box was cleaned with 70% ethanol before each trial.

*During acquisition of conditioning*, each animal received 3 pairings of a tone (85 dB, 1 kHz, 30 s) and a foot‐shock (0.3 mA, 50 Hz, 1 s). The two stimuli were separated by a trace interval of 0, 20, 40, or 60 s, depending of the group.

All mice were submitted 24 h later to the *Tone retention test* during which they were re‐exposed to the tone alone in a dark and modified chamber [2 min pre‐tone, 2 min tone, and 2 min post‐tone]. (A context test was also performed 2 h later, during which mice were re‐exposed to the conditioning chamber alone during 6 min but data are not shown, being irrelevant in the present paper).

Animals were continuously recorded for off‐line scoring of freezing, by an experimenter ignorant of the experimental condition/group. Freezing is defined as a lack of all movement except for respiratory‐related movements. Acquisition of the TFC was measured by the progression of freezing during the tone across the 3 tone deliveries. 24 h retention of the trace association was measured during the tone test by the evolution of percentage of freezing time during the tone compared to before the tone and expressed as a normalized freezing ratio: [(Tone − NoTone)/(Tone + Notone)].

### 4.4 Radial maze task of (Relational/) Declarative memory (DM)

#### 4.4.1 Apparatus

We used an open 8‐arm radial maze made of gray Plexiglas, automatized by videotracking (Imetronic). The diameter of the central platform is 30 cm, and the arms are 55 cm long by 10 cm large. Each arm is equipped with a door at its entrance and a food‐pellet delivering system at its end. The doors are individually controlled (raised up or dropped down) by the computerized system which also controls pellets availability in the food tray at the end of each arm individually, according to the task. The maze is placed in an empty room containing visual cues to enable spatial discrimination.

#### 4.4.2 Behavioral procedure

During the entire procedure, animals were submitted to one daily session. Prior to memory testing, animals were habituated to the apparatus over a period of two days during which animals were allowed to move freely in the radial maze. To complete the session, mice had to visit each arm until its end. Choice accuracy was measured as the percentage of correct responses for each pair.

##### Stage 1: Acquisition of 3‐pair discriminations

The acquisition task consisted in learning the position of the food within the maze. Indeed, each animal was assigned three adjacent pairs of arms (pairs A, B, and C). In each pair, only one arm was baited with a food reward. The experiment is designed in a way that the left arms of the pairs A and B are baited while the right arm of the pair C is. In each trial, mice were given access to a pair (either of pairs A, B, and C). A choice was considered to be made when the subject had reached the food well of an arm; this also closed the door of the non‐chosen arm. The trial was finished as soon as the animal returned to the central platform. The subject was then confined to the central platform for 20 s before the next discrimination trial began: this constituted the inter‐pair interval. Each daily session consisted of 20 consecutive trials comprising alternate presentations of pairs A, B, and C according to a pseudo‐random sequence.

A mouse was considered to reach criterion when its overall choice accuracy was at or above 75% over two consecutive sessions given that performance in each of the three discrimination choices were at least 62% correct. The test task began the following day when criterion performance was reached.

##### Stage 2: Flexibility probe of DM

In the test task, the position of the food in the maze did not change, but characteristic flexibility of DM expression was assessed by changing the way of presenting the arms. Indeed, in place of the pairs A and B, a pair AB was submitted to the mouse. The AB pair consisted in the combination of the two adjacent arms of the pairs A and B. It was the critical test of flexibility. Two other pairs were used as control: the pair C that remained unchanged (“unchanged learnt control,” and pair N (=new) made of the two arms non‐used in acquisition (“unlearnt control”).

### 4.5 Optogenetic manipulations of dCA1 activity during the radial maze DM task

*Surgery*: Mice underwent a two‐step surgery 4 weeks before the beginning of behavior. First, AAV_5_ expressing ArchT (AAV‐CaMKIIα‐ArchT‐GFP, UNC Vector Core) were bilaterally injected using glass pipettes (tip diameter 10–20 µm; 200 nl) connected to a picospritzer (Parker Hannifin Corporation) into the dCA1 at 2 injection sites to minimize diffusion to extra dCA1 areas (P_1_: AP −1.8 mm; L ± 1.3 mm; DV −1.4 mm/P_2_: AP −2.5 mm; L ± 2 mm; DV −1.4 mm, according to a classical stereotaxic procedure). Second, mice were implanted with bilateral optic fiber implants (diameter: 200 µm; numerical aperture: 0.39; flat tip; Thorlabs) directed to the dorsal dCA1 (coordinates: AP −1.8 mm, L ± 1.3 mm, and DV 1.4 mm). Implants were fixed to the skull with Super‐Bond dental cement (Sun Medical). Correct placements of fibers were visually checked on hippocampal slices to reject all mice with fiber located outside the medial part of anterior dorsal CA1.

*Optogenetic inactivation of dCA1* in aged E2‐treated mice, manipulation was performed during the acquisition phase of our radial maze task as explained in the text and Figure 3a. Briefly, the laser was ON only during the (20 s) inter‐pair intervals of each session during the acquisition stage. The light was continuously delivered (5 mW per implanted fiber) and bilaterally conducted from the laser (MGL‐FN‐526.5, CNI, China) to the mice via two fiber‐optic patch cords (diameter 200 μm, Doric Lenses), connected to a rotary joint (1 × 2 fiber‐optic rotary joint, Doric Lenses) that allowed mice to move freely in the radial maze.

### 4.6 In situ Proximity ligation assay

In order to evaluate potential redistribution of NMDARs induced by learning, the synaptic contents of GluN2(A/B)‐containing NMDARs were measured before (naive mice) and immediately after the acquisition of Trace fear conditioning (trained mice), using in situ Proximity Ligation Assay (PLA) of GluN2A/B subunits and the post‐synaptic density protein PSD‐95. PLA allows to reveal the close proximity of two proteins (Alsemarz et al., 2018; Trifilieff et al., 2011). Namely, there was a PLA signal whenever GluN2A/B was closer than 40 nm from PSD95. We use this signal as evidence of the localization of a GluN2A/B‐containing receptor at the synapse.

To confirm the specificity of the PLA signal, we performed a negative control experiment (Figure S1c–f), by replacing one of the primary antibodies of interest with the dendritic cytoskeletal protein MAP2 (Morales & Fifkova, 1989). We found little to no signal in this configuration. In contrast, the positive control experiment between PSD‐95 and the obligatory NMDA subunit GluN1 (Figure S1b) confirms the validity of the PLA signal quantified in this study.

#### 4.6.1 Brain tissue preparation

Mice were anaesthetized and perfused intracardiacally immediately after the acquisition of the trace fear conditioning, with 50 ml of ice‐cold 4% paraformaldehyde (PFA) in 0.1 M Na2HPO4/NaH2PO4 pH 7.4 buffer. Brains were post‐fixed overnight in 4% PFA at 4°C. Coronal sections (40 μm) were processed using a vibratome (Leica) and kept in a cryoprotective solution (30% glycerol, 30% ethylene glycol in PB 0.1 M pH 7.4) at −20°C until processing.

PLA was performed on floating sections using the Duolink in situ kit for brightfield detection (for main experiments) or fluorescent detection (for negative control experiments; Sigma) according to the manufacturer's instructions with the following modifications: Incubation with PLA probes was for 2 h at 37°C; ligation step was performed for 45 min at 37°C; amplification step was extended to 2 h at 37°C with a concentration of polymerase of 1/60. All antibodies were used at a concentration of 1/200 (GluN2B: Millipore 06‐600: GluN2A: alomone laboratories AGC‐002; PSD‐95: NeuroMab UC Davis 75‐028).

#### 4.6.2 Quantification of PLA signal

Brightfield or fluorescent images were acquired on a ZEISS Axio Imager A2 microscope using a 63X Oil objective. All images analyzed in this study were taken from the dorsal hippocampus of 6‐9 mice per experimental group. For all experiments, quantifications were performed from at least 8 images per animal (minimum 2 images per hemisphere; 2 slices for each animal). High‐resolution (60X 1.4 NA) images from single scans were analyzed in ImageJ (NIH) to calculate the density of PLA puncta. Images were first smoothed, and a threshold was selected manually to discriminate PLA puncta from background fluorescence. Once selected, this threshold was applied uniformly to all images in the sample set. The built‐in macro “Analyze Particles” was then used to count and characterize all objects in the thresholded images. Objects larger than 5 μm^2^ were rejected, thereby effectively removing nuclei. The remaining objects were counted as PLA puncta. Statistical analyses were performed with the StatView software (SAS institute).

### 4.7 c‐Fos Immunohistochemistry

c‐Fos immunostaining and counting was performed as previously described (Brayda‐bruno et al., 2013; c‐Fos antibody sc‐52 1/5000; Santa Cruz). Briefly, 24 h after completion of the 2 stages of the DM task, mice were exposed to a new radial maze and submitted to an acquisition session to engage their CA1. Mice were sacrificed 90 min after this training session and compared to a naive group that underwent the same manipulations except the radial maze training. For each animal, the number of c‐Fos‐immunoreactive neurons was counted bilaterally in each brain area studied (dorsal Hippocampus (CA1, CA3, DG); Dorso‐Medial Striatum (DMS); Pre‐Frontal Cortex (Infra‐Limbic (IL), Pre‐Limbic (PrL)); Lateral Entorhinal Cortex (LEC); Anterior Cingular Area (ACA)) using three to four consecutive sections. The number of positive nuclei/mm^2^ was quantified using an AxioScope A1 microscope equipped with a computerized live imaging analysis system (Mercator).

### 4.8 Microdissection and Western blot

Mice from the trace fear conditioning experiment were sacrificed two weeks after the experiment by disrupting the vertebras. The brains were cut in half, and the brain blocks containing the hippocampus was then rapidly frozen on dry ice and stored at −80°C until sectioning was done. Briefly, the frozen brain was mounted onto Tissue‐Tek^®^ OCT compound on a chuck and the brain was placed in a Leica CM3050S cryostat (Leica Microsystems) for twenty minutes to equilibrate the brain with a chamber temperature (CT) of −20°C and an object temperature (OT) of −18°C. Six, 50‐µm thick sections of the hippocampus region were cut and placed on polyethylene naphthalate (PEN) Membrane Frame slides (Carl Zeiss) under RNAase‐free conditions.

Briefly, slides were transferred from ice into ice‐cold 95% ethanol for 40 s and incubated in 75% ethanol for 30 s and in 50% ethanol for 30 s. Specimens were briefly stained in 1% cresyl violet solution. Tissue sections were dehydrated through 50% ethanol (30 s), 75% ethanol (30 s), 95% ethanol (30 s), followed by two 40 s incubation in anhydrous 100% ethanol. Slides were dried for 5 min at RT.

Immediately after dehydration, LCM was performed using a PALM MicroBeam microdissection system version 4.8 equipped with a P.A.L.M. RoboSoftware (P.A.L.M. Microlaser Technologies AG). Microdissection of CA1 apical dendrites was performed at 5× magnification. Samples were collected in adhesives caps (P.A.L.M. Microlaser Technologies AG) and were homogenized in cold lysis buffer (20 mmol/l Tris, 140 mmol/l NaCl, 3 mmol/l EDTA, 10 mmol/l NaF, 10 mmol/l Na pyrophosphate, 2 mmol/l NaVO4, 10% glycerol, pH 7.4, 1% Triton X‐100, aprotinin, leupeptin, and PMSF).

Samples were analyzed by electrophoresis (100 V, 2 h30, room temperature) through 8% polyacrylamide gels and electrophoretically transferred (60 V, 2 h30, 4°C) to nitrocellulose membranes. After overnight incubation at room temperature in Tris‐buffered saline, 5% milk and 0.05% Tween‐20, the blots were exposed to antibodies recognizing PSD‐95 (1/250; BD Bioscience 610495), GluN2A (1/10000; Millipore 5530), or GluN2B (1/250; Transduction labs, 610417 BD Bioscience) antibody overnight at 4°C. The primary antibodies were revealed using the corresponding mice or rabbit peroxidase‐conjugated secondary antibodies (1/2000) for 1 h at room temperature. Peroxidase activity was detected using the Santa Cruz chemiluminescence kit. Results were normalized to GAPDH expression and expressed as a fold change (%) to young vehicle.

### 4.9 Dendritic spine density analysis

Golgi–Cox staining was performed using the FD Rapid GolgiStain kit according to manufacturer's instructions (FD NeuroTechnologies). Mice were sacrificed by cervical dislocation and brains rapidly removed, rinsed briefly in water and processed according to the manufacturer's protocol. Sections of 100 μm were obtained with a Leica VT1200S vibratome (Leica Microsystems). Sections of 150 μm were obtained with a Leica VT1200S vibratome (Leica Microsystems). Images of CA1 stratum radiatum were taken using a Zeiss microscope at ×63 magnification and then spines were measured using ImageJ software. Protuberance with a height <1.8 µm were classified as spines. Spines morphology was classified according to spine's head diameter: above 0.6 µm spines were classified as mushrooms, and below 0.6 µm, spines were classified as thin (Nodé‐Langlois et al., 2006).

### 4.10 Interfering peptide

#### 4.10.1 Surgery

Mice were anaesthetized with isoflurane (isoflurane concentration: 1.5%–2%, O_2_ flow rate: 11/min) and secured in a stereotaxic frame (Kopf Instruments). Stainless‐steel guide‐cannulae (26 gauge, 8 mm length) were implanted bilaterally 1 mm above the stratum pyramidale of CA1 in dorsal hippocampus (A/P, −2 mm; M/L, ±1.3 mm; D/V, −1 mm; relative to bregma), then fixed in place with two jewel screws attached to the skull and dental cement. Mice were then allowed to recover in their home cage for at least 10 days before the behavioral experiment.

#### 4.10.2 Peptides

The Tat‐NR2B‐9c peptide disrupts GluN2B/PSD‐95 interaction (Figure 1d, Figure S2a) but not GluN2A/PSD‐95 (Aarts et al., 2002; Gardoni et al., 2009). The peptide comprises the nine COOH‐terminal residues of GluN2B subunit (Lys‐Leu‐Ser‐Ser‐Ile‐Glu‐Ser‐Asp‐Val; Clinisciences) in order to bind PDZ2 domain of PSD‐95. Tat‐NR2B‐9c peptide is rendered cell‐permeant by fusing it to the cell‐membrane transduction domain of the human immunodeficiency virus‐type 1 (HIV‐1), the Tat protein (Tyr‐Gly‐Arg‐Lys‐Lys‐Arg‐Arg‐Gln‐Arg‐Arg‐Arg) to obtain a 20 –amino acid peptide Tat‐NR2B‐9c. The control peptide Tat‐NR2B‐AA contains a double point mutation (Lys‐Leu‐Ser‐Ser‐Ile‐Glu‐Ala‐Asp‐Ala; Tat‐NR2B‐AA) and cannot bind to the PDZ2 domain of PSD‐95 (Aarts et al., 2002). Tat‐NR2B‐AA was synthesized and provided by Peter Vanhoutte.

### 4.11 Intra‐dCA1 infusion in awake animals

Stainless‐steel *cannulae* (32 gauge, 9 mm length) attached to 1‐µl Hamilton syringes with polyethylene catheter tubing were inserted through the guide‐*cannulae*. The syringes were fixed in a constant rate infusion pump (Harvard Apparatus, 0.1 µl/min). Animals received intra‐dCA1 (Figure 1d) bilateral infusions (0.3 µl per side (Potier et al., 2016), 41.6 µg/L (Cahill et al., 2014)) of Tat‐NR2B‐9c peptide, Tat‐NR2B‐AA peptide, or artificial cerebrospinal fluid (aCSF) 1 h before the acquisition of trace fear conditioning. The *cannulae* were left in place for 2 min before removal to allow diffusion of peptides away from the *cannulae* tips. Such timing was chosen (a) in order to match previous experiments (Potier et al., 2016), showing, respectively, beneficial or deleterious effects on memory of E2 or crosslink of NMDA receptors, injected in the dCA1 1 h before training and (b) because 1 h is within the peptide's efficacy window [estimated half‐life of the TAT‐GluN2b‐9C of 2.8 h, according to the ExPaSy resource (https://web.expasy.org/protparam/) and unpublished data showed that the TAT‐GluN2b‐9C peptide was efficient between 30 min and 4 h after application]. The dose of peptide injected was determined on the basis of the literature and pilot experiment showing no memory enhancing effect of a lower dose.

#### 4.11.1 Control experiment

*Confirming the efficacy of the TAT*‐*NR2B*‐*9c* to disrupt the interaction between GluN2B‐containing NMDAR and PSD‐95 and hence decreasing GluN2B synaptic contents. Aged mice were bilaterally injected using glass pipettes (tip diameter 10–20 µm; 0.3 µl/side) connected to a picospritzer (Parker Hannifin Corporation) into the dCA1 (AP −1.8 mm; L ± 1.3 mm; DV −1.4 mm). Mice were then sutured and placed back into their homecage. 1 h later, they were intracardiacally perfused with 4% PFA and PLA staining/quantification was performed as described above. Results are described in Figure S2a.

### 4.12 Statistical analyses

All statistical analyses were carried out using the Statview software.

Behavioral data were submitted to either one‐way analysis of variance (ANOVA) with the between‐subject factor: Age or treatment, or two‐way ANOVA to test the interaction between age/treatment and repeated measures of performance/freezing (for the acquisition of *trace fear conditioning* and *radial* *maze task*, and for the evolution between the acquisition phase and the test phase of the radial maze task). Post hoc comparisons were performed using Fisher PLSD test.

Molecular (PLA), immunohistochemical (Fos) and Golgi data were analyzed by *t* tests comparing the experimental group to its control group.

Data are presented as means ± *SEM*, with *p < 0.05; **p < 0.01; ***p < 0.001.

## CONFLICT OF INTEREST

All co‐authors reported no biomedical financial interests or potential conflicts of interest.

## AUTHOR CONTRIBUTIONS

A.S.A., P.T., P.V, C.B.‐P., and A.M. designed research. A.S.A., A.S., E.G.D., P.G.‐L., L.B.‐B., V.L., and M.P. performed research. P.V contributed to new reagents/analytic tools. A.S.A, A.S., P.T., M.P., and A.M. analyzed the data. A.S.A., P.T., A.D., P.V, N.S., C.B.‐P., and A.M. discussed the results. A.S.A., P.T., A.D., P.V., M.P., and A.M. wrote the paper.

## Supporting information

Fig S1‐S3

## Data Availability

Data will be made available upon reasonable request.

## References

[acel13243-bib-0001] Aarts, M. , Liu, Y. , Liu, L. , Besshoh, S. , Arundine, M. , Gurd, J. W. , Wang, Y.‐T. , Salter, M. W. , & Tymianski, M. (2002). Treatment of ischemic brain damage by perturbing NMDA receptor‐ PSD‐95 protein interactions. Science, 298, 846–850. 12399596 10.1126/science.1072873

[acel13243-bib-0002] Al Abed, A. S. , Sellami, A. , Brayda‐Bruno, L. , Lamothe, V. , Noguès, X. , Potier, M. , Bennetau‐Pelissero, C. , & Marighetto, A. (2016). Estradiol enhances retention but not organization of hippocampus‐dependent memory in intact male mice. Psychoneuroendocrinology, 69, 77–89. 27038677 10.1016/j.psyneuen.2016.03.014

[acel13243-bib-0003] Alsemarz, A. , Lasko, P. , & Fagotto, F. (2018). Limited significance of the in situ proximity ligation assay. bioRxiv, 411355.

[acel13243-bib-0004] Baudry, M. , Zhu, G. , Liu, Y. , Wang, Y. , Briz, V. , & Bi, X. (2015) Multiple cellular cascades participate in long‐term potentiation and in hippocampus‐dependent learning. Brain Research, 1621, 73–81. 25482663 10.1016/j.brainres.2014.11.033 PMC4532652

[acel13243-bib-0005] Bean, L. A. , Ianov, L. , & Foster, T. C. (2014). Estrogen receptors, the hippocampus, and memory. Neuroscience, 20, 534–545. 10.1177/1073858413519865 PMC4317255 24510074

[acel13243-bib-0006] Bellone, C. , & Nicoll, R. A. (2007). Rapid bidirectional switching of synaptic NMDA receptors. Neuron, 55, 779–785. 17785184 10.1016/j.neuron.2007.07.035

[acel13243-bib-0007] Brayda‐bruno, L. , Mons, N. , Yee, B. K. , Micheau, J. , Abrous, D. N. , Nogues, X. , & Marighetto, A. (2013). Partial loss in septo‐hippocampal cholinergic neurons alters memory‐dependent measures of brain connectivity without overt memory deficits. Neurobiology of Disease, 54, 372–381. 10.1016/j.nbd.2013.01.010 23376311

[acel13243-bib-0008] Cahill, E. , Pascoli, V. , Trifilieff, P. , Savoldi, D. , Kappès, V. , Lüscher, C. , Caboche, J. , & Vanhoutte, P. (2014). D1R/GluN1 complexes in the striatum integrate dopamine and glutamate signalling to control synaptic plasticity and cocaine‐induced responses. Molecular Psychiatry, 19, 1295–1304. 25070539 10.1038/mp.2014.73 PMC4255088

[acel13243-bib-0009] Dupuis, J. P. , Ladepeche, L. , Seth, H. , Bard, L. , Varela, J. , Mikasova, L. , Bouchet, D. , Rogemond, V. , Honnorat, J. , Hanse, E. , & Groc, L. (2014). Surface dynamics of GluN2B‐NMDA receptors controls plasticity of maturing glutamate synapses. EMBO Journal, 33, 842–861. 10.1002/embj.201386356 24591565 PMC4194110

[acel13243-bib-0010] Eichenbaum, H. , & Cohen, N. J. (2014). Can we reconcile the declarative memory and spatial navigation views on hippocampal function? Neuron, 83, 764–770. 25144874 10.1016/j.neuron.2014.07.032 PMC4148642

[acel13243-bib-0011] Etchamendy, N. , Konishi, K. , Pike, G. B. , Marighetto, A. , & Bohbot, D. (2012). Evidence for a virtual human analog of a rodent relational memory task: A study of aging and fMRI in young adults. Hippocampus, 22, 869–880. 21656872 10.1002/hipo.20948

[acel13243-bib-0012] Frick, K. M. (2015). Molecular mechanisms underlying the memory‐enhancing effects of estradiol. Hormones and Behavior, 74, 4–18. 10.1016/j.yhbeh.2015.05.001 25960081 PMC4573242

[acel13243-bib-0013] Gardoni, F. , Mauceri, D. , Malinverno, M. , Polli, F. , Costa, C. , Tozzi, A. , Siliquini, S. , Picconi, B. , Cattabeni, F. , Calabresi, P. , & Di Luca, M. (2009). Decreased NR2B subunit synaptic levels cause impaired long‐term potentiation but not long‐term depression. Journal of Neuroscience, 29, 669–677. 19158293 10.1523/JNEUROSCI.3921-08.2009 PMC6665154

[acel13243-bib-0014] Hara, Y. , Crimins, J. L. , Puri, R. , Wang, A. C. J. , Motley, S. E. , Yuk, F. , Ramos, T. M. , Janssen, W. G. M. , Rapp, P. R. , & Morrison, J. H. (2018). Estrogen alters the synaptic distribution of phospho‐GluN2B in the dorsolateral prefrontal cortex while promoting working memory in aged rhesus monkeys. Neuroscience, 394, 303–315. 10.1016/j.neuroscience.2018.09.021 30482274 PMC6394848

[acel13243-bib-0015] Huerta, P. T. , Sun, L. D. , Wilson, M. A. , & Tonegawa, S. (2000). Formation of temporal memory requires NMDA receptors within CA1 pyramidal neurons. Neuron, 25(2), 473–480. 10.1016/S0896-6273(00)80909-5 10719900

[acel13243-bib-0016] Kellermayer, B. , Ferreira, J. S. , Dupuis, J. , Levet, F. , Grillo‐Bosch, D. , Bard, L. , Linarès‐Loyez, J. , Bouchet, D. , Choquet, D. , Rusakov, D. A. , Bon, P. , Sibarita, J.‐B. , Cognet, L. , Sainlos, M. , Carvalho, A. L. , & Groc, L. (2018). Differential nanoscale topography and functional role of GluN2‐NMDA receptor subtypes at glutamatergic synapses. Neuron, 100(1), 106–119.e7. 10.1016/j.neuron.2018.09.012 30269991

[acel13243-bib-0017] Kumar, A. , Thinschmidt, J. S. , & Foster, T. C. (2019). Subunit contribution to NMDA receptor hypofunction and redox sensitivity of hippocampal synaptic transmission during aging. Aging, 11(14), 5140–5157. 10.18632/aging.102108 31339863 PMC6682512

[acel13243-bib-0018] Lewis, M. C. , Kerr, K. M. , Orr, P. T. , & Frick, K. M. (2008). Estradiol‐induced enhancement of object memory consolidation involves NMDA receptors and protein kinase A in the dorsal hippocampus of female C57BL/6 mice. Behavioral Neuroscience, 122(3), 716–721. 10.1037/0735-7044.122.3.716 18513142 PMC2673328

[acel13243-bib-0019] Marighetto, A. , Etchamendy, N. , Touzani, K. , Torrea, C. C. , Yee, B. K. , Rawlins, J. N. P. , & Jaffard, R. (1999). Knowing which and knowing what: a potential mouse model for age‐related human declarative memory decline. European Journal of Neuroscience, 11, 3312–3322. 10510196 10.1046/j.1460-9568.1999.00741.x

[acel13243-bib-0020] Morales, M. , & Fifkova, E. (1989). Distribution of MAP 2 in dendritic spines and its colocalization with actin. Cell and Tissue Research, 256(3), 447–456. 10.1007/BF00225592 2743390

[acel13243-bib-0021] Nicoll, R. (2017). A brief history of long‐term potentiation. Neuron, 93, 281–290. 28103477 10.1016/j.neuron.2016.12.015

[acel13243-bib-0022] Niethammer, M. , Kim, E. , & Sheng, M. (1996). Interaction between the C terminus of NMDA receptor subunits and multiple members of the PSD‐95 family of membrane‐associated guanylate kinases. Journal of Neuroscience, 16, 2157–2163. 8601796 10.1523/JNEUROSCI.16-07-02157.1996 PMC6578538

[acel13243-bib-0023] Nodé‐Langlois, R. , Muller, D. , & Boda, B. (2006). Sequential implication of the mental retardation proteins ARHGEF6 and PAK3 in spine morphogenesis. Journal of Cell Science, 119, 4986–4993. 17105769 10.1242/jcs.03273

[acel13243-bib-0024] Paoletti, P. , Bellone, C. , & Zhou, Q. (2013). NMDA receptor subunit diversity: Impact on receptor properties, synaptic plasticity and disease. Nature Reviews Neuroscience, 14, 383–400. 23686171 10.1038/nrn3504

[acel13243-bib-0025] Potier, M. , Georges, F. , Brayda‐Bruno, L. , Ladépêche, L. , Lamothe, V. , Al Abed, A. S. , Groc, L. , & Marighetto, A. (2016). Temporal memory and its enhancement by estradiol requires surface dynamics of hippocampal CA1 N‐Methyl‐D‐aspartate receptors. Biological Psychiatry, 79, 735–745. 26321020 10.1016/j.biopsych.2015.07.017

[acel13243-bib-0026] Quinlan, E. M. , Lebel, D. , Brosh, I. , & Barkai, E. (2004). A molecular mechanism for stabilization of learning‐induced synaptic modifications. Neuron, 41, 185–192. 14741100 10.1016/s0896-6273(03)00874-2

[acel13243-bib-0027] Sellami, A. , Abed, A. , Brayda‐Bruno, L. , Etchamendy, N. , Valério, S. , Oulé, M. , Pantaléon, L. , Lamothe, V. , Potier, M. , Bernard, K. , Jabourian, M. , Herry, C. , Mons, N. , Marighetto, A. (2018). Protocols to study declarative memory formation in mice and humans: Optogenetics and translational behavioral approaches. Bio‐protocol, 8(12). 10.21769/bioprotoc.2888 PMC8275238 34285997

[acel13243-bib-0028] Sellami, A. , Al Abed, A. S. , Brayda‐Bruno, L. , Etchamendy, N. , Valério, S. , Oulé, M. , Pantaléon, L. , Lamothe, V. , Potier, M. , Bernard, K. , Jabourian, M. , Herry, C. , Mons, N. , Piazza, P.‐V. , Eichenbaum, H. , & Marighetto, A. (2017). Temporal binding function of dorsal CA1 is critical for declarative memory formation. Proceedings National Academy of Science of the United States of America, 114, 10262–10267. 10.1073/pnas.1619657114 PMC5617244 28874586

[acel13243-bib-0029] Shipton, O. A. , & Paulsen, O. (2014). GluN2A and GluN2B subunit‐containing NMDA receptors in hippocampal plasticity. Philosophical Transactions of the Royal Society of London. Series B, Biological Sciences, 369, 20130163. 24298164 10.1098/rstb.2013.0163 PMC3843894

[acel13243-bib-0030] Smith, C. C. , & McMahon, L. L. (2006). Estradiol‐induced increase in the magnitude of long‐term potentiation is prevented by blocking NR2B‐containing receptors. Journal of Neuroscience, 26, 8517–8522. 16914677 10.1523/JNEUROSCI.5279-05.2006 PMC6674362

[acel13243-bib-0031] Taylor, A. M. , Bus, T. , Sprengel, R. , Seeburg, P. H. , Rawlins, J. N. P. , & Bannerman, D. M. (2014). Hippocampal NMDA receptors are important for behavioural inhibition but not for encoding associative spatial memories. Philosophical Transactions of the Royal Society B: Biological Sciences, 369(1633), 20130149. 10.1098/rstb.2013.0149 PMC3843881 24298151

[acel13243-bib-0032] Trifilieff, P. , Rives, M.‐L. , Urizar, E. , Piskorowski, R. , Vishwasrao, H. , Castrillon, J. , Schmauss, C. , Slättman, M. , Gullberg, M. , & Javitch, J. (2011). Detection of antigen interactions ex vivo by proximity ligation assay: Endogenous dopamine D2‐adenosine A2A receptor complexes in the striatum. BioTechniques, 51, 111–118. 21806555 10.2144/000113719 PMC3642203

[acel13243-bib-0033] Tsien, J. Z. , Huerta, P. T. , & Tonegawa, S. (1996). The essential role of hippocampal CA1 NMDA receptor – Dependent synaptic plasticity in spatial memory. Cell, 87, 1327–1338. 8980238 10.1016/s0092-8674(00)81827-9

[acel13243-bib-0034] Vedder, L. C. , Smith, C. C. , Flannigan, A. E. , & McMahon, L. L. (2013). Estradiol‐induced increase in novel object recognition requires hippocampal NR2B‐containing NMDA receptors. Hippocampus, 23(1), 108–115. 10.1002/hipo.22068 22965452 PMC4038023

[acel13243-bib-0035] Wang, D. , Jacobs, S. A. , & Tsien, J. Z. (2014). Targeting the NMDA receptor subunit NR2B for treating or preventing age‐related memory decline. Expert Opinion on Therapeutic Targets, 18(10), 1121–1130. 10.1517/14728222.2014.941286 25152202

[acel13243-bib-0036] Waters, E. M. , Mazid, S. , Dodos, M. , Puri, R. , Janssen, W. G. , Morrison, J. H. , McEwen, B. S. , & Milner, T. A. (2019). Effects of estrogen and aging on synaptic morphology and distribution of phosphorylated Tyr1472 NR2B in the female rat hippocampus. Neurobiology of Aging, 73, 200–210. 10.1016/j.neurobiolaging.2018.09.025 30384123 PMC11548941

[acel13243-bib-0037] Woolley, C. S. , & Mcewen, B. S. (1994). Estradiol regulates hippocampal dendritic spine density via an an N‐methyl‐D‐aspartate receptor‐dependent mechanism. Journal of Neuroscience, 14, 7680–7687. 7996203 10.1523/JNEUROSCI.14-12-07680.1994 PMC6576901

[acel13243-bib-0038] Yashiro, K. , & Philpot, B. D. (2008). Regulation of NMDA receptor subunit expression and its implications for LTD, LTP, and metaplasticity. Neuropharmacology, 55(7), 1081–1094. 10.1016/j.neuropharm.2008.07.046 18755202 PMC2590778

[acel13243-bib-0039] Zamzow, D. R. , Elias, V. , Shumaker, M. , Larson, C. , & Magnusson, K. R. (2013). An increase in the association of GluN2B containing NMDA receptors with membrane scaffolding proteins was related to memory declines during aging. Journal of Neuroscience, 33(30), 12300–12305. 10.1523/JNEUROSCI.0312-13.2013 23884936 PMC3721840

