# Supplementary material for: Age‐related impairment of declarative memory: linking memorization of temporal associations to GluN2B redistribution in dorsal CA1
Source: Aging Cell. 2020 Oct 3;19(10):e13243. doi: 10.1111/acel.13243 (PMC7576225; doi:10.1111/acel.13243)
Supplement: Supplementary file 1 — Fig S1‐S3 [file ACEL-19-e13243-s001.docx]

**
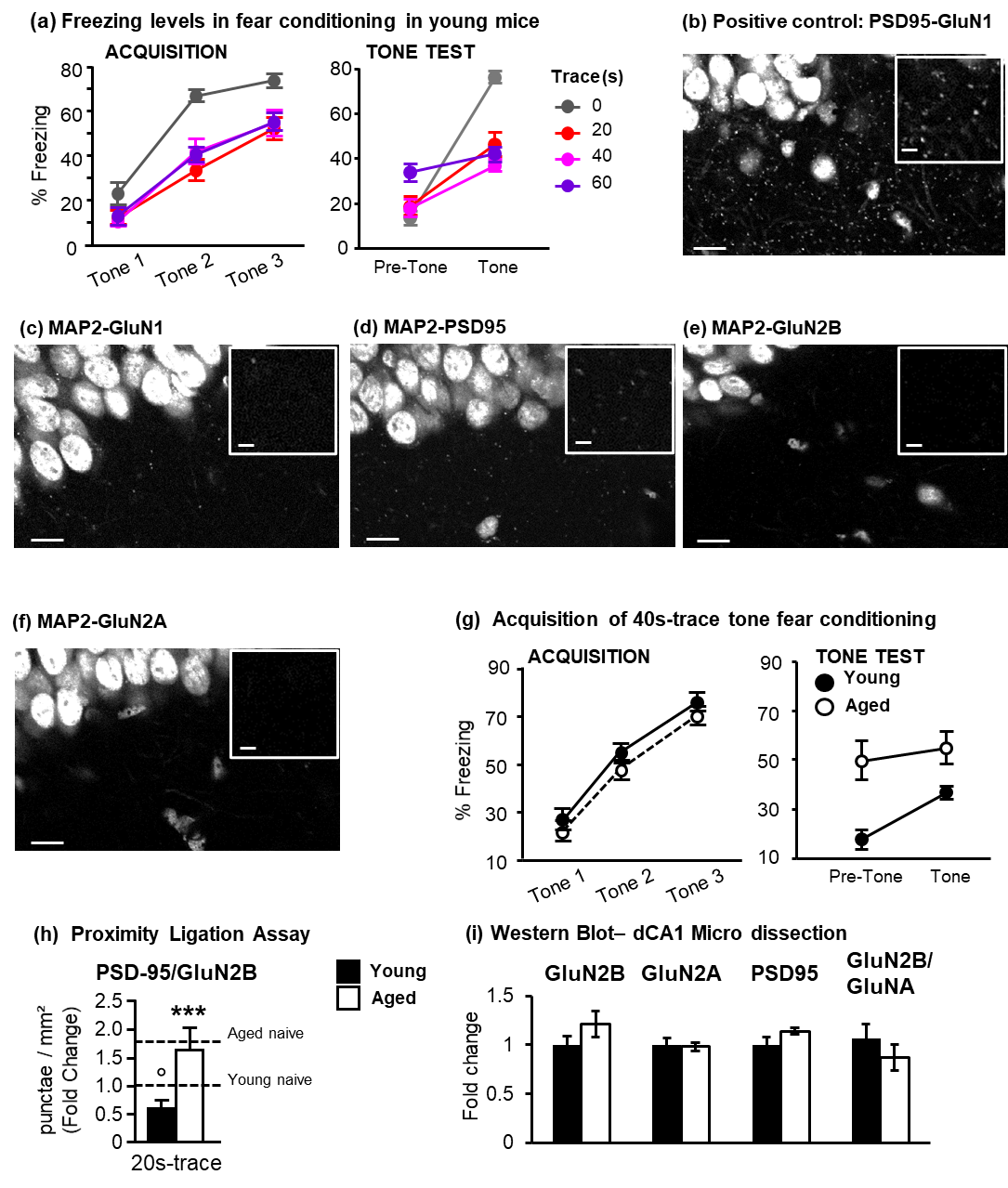
Supplementary figures**

**Figure S1: Supplementary data to behavioral and molecular findings presented in Figure 1 demonstrating that the extra-synaptic redistribution of GluN2B-NMDAR induced by the acquisition of trace tone fear conditioning specifically correlates with successful memorization of the trace association (i.e. efficient temporal binding). (a) *Acquisition of (0-60 s-) trace tone fear conditioning in young mice:*** showing that the progression of percentage of freezing during the tone across its successive presentations during conditioning (3 tone-shock pairings) was similar among all groups trained with a trace> 0, whereas only the groups trained with a trace <60s, exhibited significant retention of the trace-tone-shock association and significant reduction of GluN2B synaptic contents after acquisition of conditioning relative to the naive condition (**Figure 1b**). Therefore the diminution of GluN2B synaptic contents cannot be due to unspecific effect of stress (freezing levels during and at the end of conditioning are similar among the trace groups) and is truly associated to efficient memorization of the association between discontiguous stimuli, i.e. efficient TB function. **Right panel** shows percentages of time spent freezing before and during tone delivery in the Tone Test (same results are represented as freezing ratio in Figure 1b). **(b-f) fluorescent labeling of PLA signal of positive (b; NR1-PSD95 interaction) and negative (c-f) control.** For the negative controls, we used the cytoskeleton associated protein MAP2 in place of one of the proteins of interest. As illustrated, no PLA signal can be observed when performing Proximity Ligation Assays between MAP2 and NMDAR/PSD-95, unlike for GluN1-PSD95 (b). This further support the validity of our quantifications of the NMDAR subunit GluN2A/B synaptic content using PLA. Scale bar: 10 µm. Inset shows a zoomed portion of the image. **(g) *Acquisition of 40 s-trace tone fear conditioning*** similarly shows no effect of age on freezing levels and their evolution during conditioning, excluding unspecific effect of stress as explanation for the age-related differences observed on both retention of the trace-association and GluN2B synaptic contents (cf **Figure 1c**). **Right panel** shows percentages of freezing before and during tone delivery in the Tone Test (same results are represented as ratio in **Figure 1c)**. **(h) *Proximity ligation assay*** revealing PSD-95/GluN2B complexes following the acquisition of 20s-trace tone fear conditioning in young and aged mice, confirms the age-related increase in GluN2B synaptic contents and alteration of learning-induced redistribution of these receptors outside the post-synaptic densities **(**seen under the 40s-trace condition **Figure 1c)**. **(i) *Western blots of GluN2B, GluN2A, and PSD95 performed on micro-dissected samples of dCA1*** show no effect of age on expression of these proteins, suggesting that age-related differences revealed by PLA measures of PSD95/GluN2B interaction reflect alteration in the trafficking of GluN2B-NMDAR between the synaptic and extra-synaptic compartments. Data presented as mean ± SEM. ***: p≤0.001 young vs aged, °: p<0.05 vs naive condition.

**
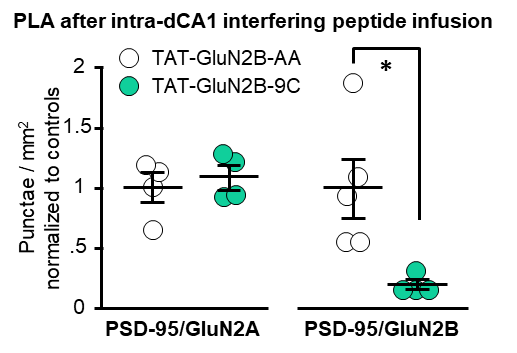
Figure S2: Control experiments of the effect of the interfering peptide.** Quantifications of the PLA signal of GluN2A-PSD95 (left) and GluN2B-PSD95 (right) after intra-dCA1 infusion of the interfering peptide TAT-GluN2B-9c and its control TAT-GluN2B-AA in aged mice. Levels of punctea / mm^2^ in the TAT-GluN2B-9c group are normalized to the control- (TAT-GluN2B-AA) injected group. Infusion of the interfering peptide TAT-GluN2B-9c successfully decreased the synaptic content of GluN2B-containing NMDAR (*TAT-GluN2B-9c vs TAT-GluN2b-AA:* p = 0.0288; t-test), while leaving the levels of GluN2A subunit unaffected. Data presented as mean ± SEM. *: p≤0.05.

**
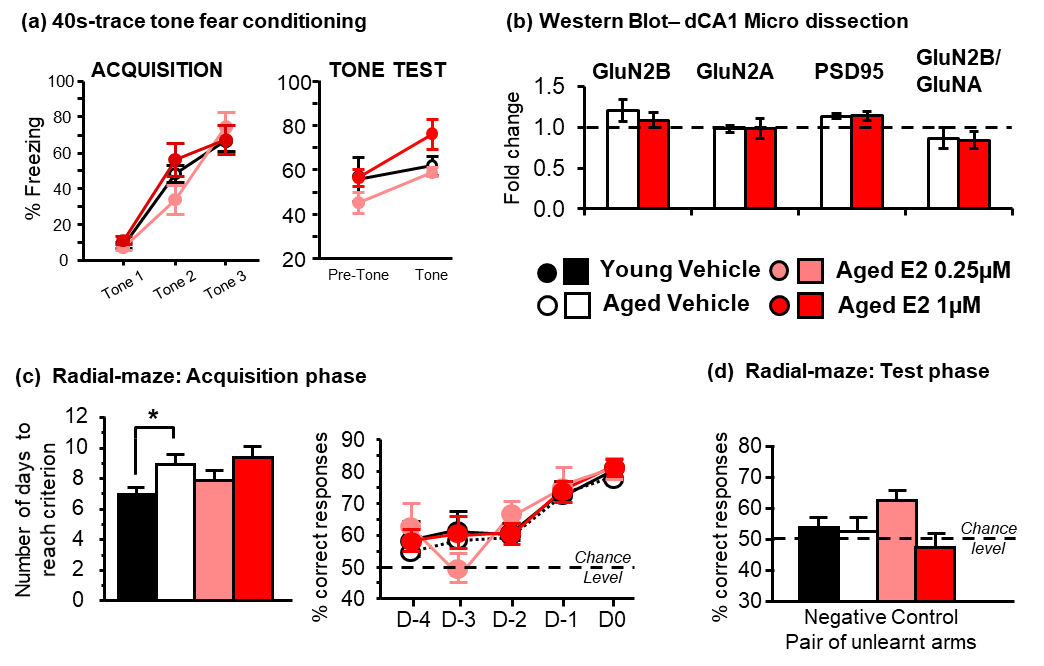
**

**Figure S3: Supplementary data to behavioral and molecular findings presented in Figure 2 showing the specificity of beneficial effects of supplementation with 17β-estradiol (E2) at 1µM (in drinking-water) on the impairments of temporal binding-dependent memory and of GluN2B-NMDAR trafficking in aged mice.**

**(a) left panel:** The percentage of freezing during tone presentation and its evolution of across successive tone presentations during conditioning was similar among the aged groups, showing that the treatment with E2 had no effect on the acquisition of 40s-trace tone fear conditioning. This observation excludes unspecific effect of stress as explanation for E2 1µM-induced normalization of retention of the trace-association and GluN2B synaptic contents in aged mice (**Figure 2a**). **Right panel:** percentage of freezing in the Tone Test (**Figure 2a**). **(b) *Western blots of GluN2B, GluN2A, and PSD95 performed on micro-dissected samples of dCA1, as well as GluN2A/GluN2b ratio*** showed no effect of E2 1µM on expression of these proteins, suggesting that its effect on PLA measures of PSD95/GluN2B interaction reflects normalization of the trafficking of GluN2B-NMDAR between the synaptic and extra-synaptic compartments in aged mice. **(c-d) In the radial-maze task of declarative memory** the treatment had no effect on performance neither in the acquisition of the 3 pair discrimination [i.e. no differences in the number of days to reach the learning criterion **(c)** **left panel**, percentages of correct response in the last 5 days of acquisition **(c)** **right panel,** nor in the negative control pair of the test phase **(d)**]. Thus, E2 1µM selectively improved the age-associated impairment of declarative memory flexibility (**Figure 2b**: flexibility probe). Data presented as mean ± SEM. *: p≤0.05.
